# A neglected point in surgical treatment of adolescent idiopathic scoliosis: Variations in the number of vertebrae

**DOI:** 10.1097/MD.0000000000004682

**Published:** 2016-08-26

**Authors:** Zongshan Hu, Zhen Zhang, Zhihui Zhao, Zezhang Zhu, Zhen Liu, Yong Qiu

**Affiliations:** Department of Spine Surgery, The Affiliated Drum Tower Hospital of Nanjing University Medical School, Nanjing, China.

**Keywords:** adolescent idiopathic scoliosis, thoracic–lumbar vertebrae, variation of vertebral number

## Abstract

Inaccurate identification of vertebral levels is the main cause of wrong-site spine surgery which is performed by nearly half of the spine surgeons. Unusual anatomy and failure to verify the surgical level on radiographs have been commonly reported. We aimed at investigating the variations in vertebral number in adolescent idiopathic scoliosis (AIS) patients and thus to raise awareness of the possibility for wrong-level spinal surgery and to make a comparison with normal adolescents. A cohort of 657 AIS patients and 248 normal adolescents, presented to our center from June 2008 to February 2013, who met the inclusion criteria, were recruited. Radiographs were reviewed to identify the number of thoracic or lumbar vertebrae and the presence of a lumbosacral transitional vertebra. In the AIS group, 70 (10.6%) patients had variations in the number of thoracic and/or lumbar vertebrae. Remarkably, the prevalence of variations in male subjects was significantly higher than that in female subjects (*P* < 0.05). Thirty-seven patients (5.6%) had an atypical number of thoracic vertebrae, with 33 having 11 thoracic vertebrae and 4 patients having 13. Forty-eight patients (7.3%) had an atypical number of lumbar vertebrae, with 14 having 4 lumbar vertebrae and 34 patients having 6. Multilevel vertebral anomalies were present in 2.3% of the patients (15 of 657). A variation in the number of vertebrae had been identified in 1.7% (11) of the reports by the radiologist. In the normal group, 27 (10.9%) subjects showed variations in the vertebral number. There was no significant difference in the prevalence of atypical numbers of vertebral number between the AIS and normal groups (*P* > 0.05). Therefore, we concluded that variations in the number of thoracic–lumbar vertebrae were found in up to10.6% of AIS patients. Identification of variations in the number of vertebrae is crucial to serve to decrease the risk of wrong-level surgery.

## 1 Introduction

Scoliosis is a structural, lateral, and rotated curvature of the spine. Adolescent idiopathic scoliosis (AIS) is a kind of scoliosis that arises in otherwise healthy children at or around puberty with an incidence of 1% to 4.5%.^[1–4]^ The incidence of AIS were 3.26% and 4.5% in Korea and Canada, respectively.^[2,3]^ The options for treating AIS include observation, bracing, and surgery. The goals in scoliosis surgery are to reduce curvature and to create a stable framework on which vertebral fusion can occur.^[1,5–9]^ Unfortunately, there is a high prevalence of wrong-level surgery performed by spine surgeons.^[10]^ Nearly half of surveyed spine surgeons responded that they had performed surgery at an incorrect vertebral level during their careers.^[11]^ Unusual patient anatomy and a failure to verify the operative site on radiography have been commonly reported.^[10,11]^ Therefore, it is necessary to count thoracic–lumbar vertebrae preoperatively to avoid wrong-level surgery in the course of scoliosis correction due to the variation in vertebral number. Recently, Ibrahim et al^[12]^ reported for the first time that variations in the numbers of thoracic or lumbar vertebrae were found in 10% of patients with AIS. However, there has been a lack of identification of sex-dependent differences and of the use of normal subjects as a control group.

It has been widely reported that the prevalence of numerical variations in vertebrae has varied in different geographic areas and in different races (Table 1), but few studies have considered the presence of 13 thoracic vertebrae and 4 lumbar vertebrae in 1 person or 11 thoracic vertebrae and 6 lumbar vertebrae in another, for a total of 17 thoracic–lumbar vertebrae, as a numerical variation in the number of vertebrae.^[13–17]^

**Table 1:**
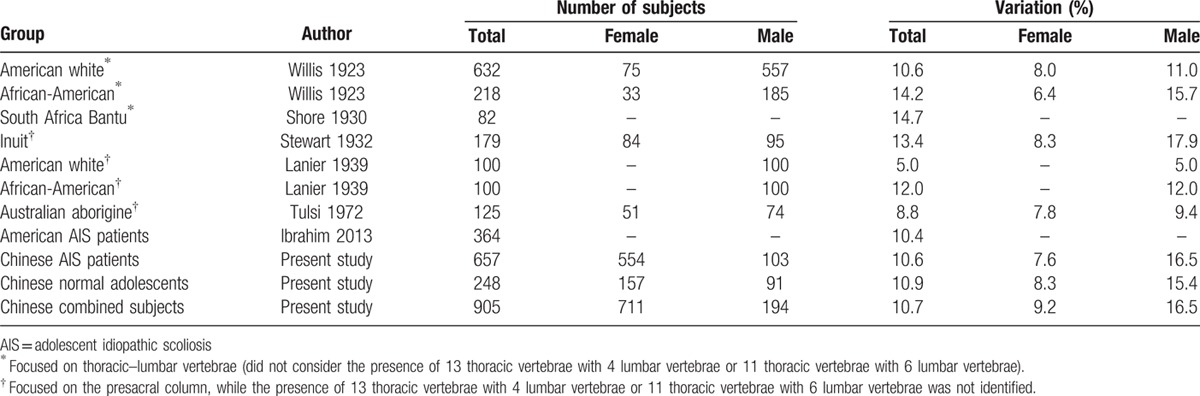
A comparison of variations in vertebral number.

Considering the variations of vertebral number reported in different races and American AIS patients but related studies rarely being reported in China, it is necessary to analyze the variations of vertebral number in the Chinese patients with AIS. The objective of this study was to ascertain the incidence of variations in the number of thoracic–lumbar vertebrae in patients with AIS and thus to raise awareness of the possibility for wrong-level spinal surgery, as well as to make a comparison with normal adolescents. Moreover, the variations in the number of vertebra were investigated through comparison between the current cohort of subjects and another cohort with different ethnicity background in previous studies.^[12,14,16]^

## 2 Patients and methods

### 2.1 Patients

**Figure 1:**
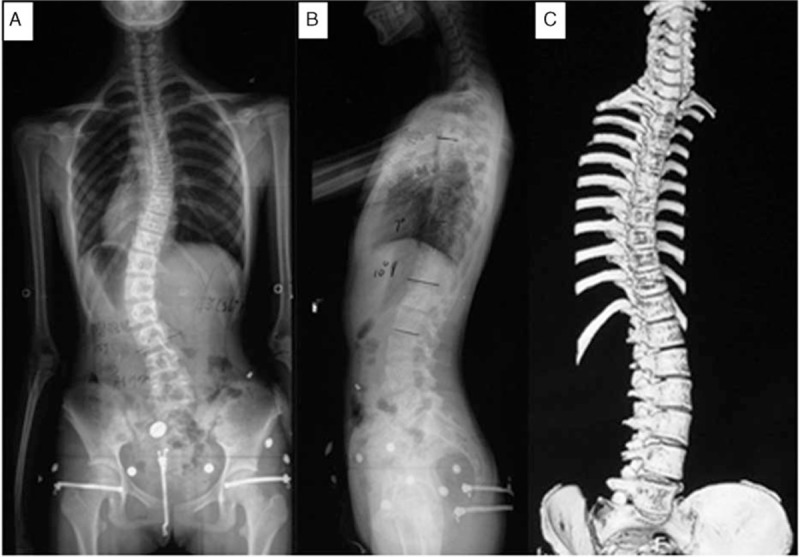
A 16-year-old female patient with adolescent idiopathic scoliosis. Preoperative thoracic and lumbar curve were 36° and 40°, respectively. Eleven thoracic vertebrae and 6 lumbar vertebrae was found on the full-spine anteroposterior and lateral plain films (A and B) and determined by the 3D-reconstruction preoperatively (C).

This retrospective study was approved by the Ethics Committee of our hospital. Written informed consent was not obtained from patients in this study because we anonymized and deidentified the patients’ information before analysis. A cohort of 692 patients with AIS from the Han Chinese population who underwent scoliosis surgery in our center were included in this study from June 2008 to February 2013. Inclusion criteria were as follows: aged between 10 and 18 years, preoperative Cobb angle more than 40°, complete radiographs including standing anteroposterior (AP) and lateral spine views, and computed tomography 3-dimensional reconstruction images (Fig. 1). Exclusion criteria included patients with neuromuscular diseases, previous history of spine surgery, or incomplete radiographs. Another 261 healthy adolescents were recruited as the normal group. These normal subjects, who presented to our center because of complaints of minor back discomfort from June 2008 to February 2013, were found to have normal spinal alignment on standing AP and lateral radiographs of the whole spine.

On the AP view, the vertebra to which the most cephalad rib was attached was identified as the first thoracic level. All vertebrae with corresponding rib attachments were counted as thoracic vertebrae, regardless of complete, vestigial, hypoplastic, bilateral, or unilateral ribs; a vertebral level was counted as a lumbar vertebral level only if there was no rib attachment. The first lumbar vertebra was the vertebra following the last enumerated thoracic vertebra.^[12]^ A lumbosacral transitional vertebra (LSTV) was considered a lumbar vertebra when the transverse processes attached to the sacrum through incomplete or complete osseous fusion or a diarthrodial joint.^[18]^ All the radiographs were reviewed by 2 residents in our center.

### 2.2 Statistical analysis

The data were analyzed using SPSS software, version 19.0 (SPSS Inc., Chicago, IL). The chi-square tests with Fisher exact probability were performed to compare age, gender ratio, and the incidence of variations in the numbers of thoracic–lumbar vertebrae between the 2 groups. The chi-square test was also employed to compare the ethnicity-related variations in the number of vertebra. Sensitivity analyses were conducted by the following equation: sensitivity = A/(A + C) × 100%. All *P* values were 2-sided with *P* < 0.05 considered to be statistically significant. In addition, the ethnicity-related differences of the variations in the number of vertebra were investigated through comparison between the current AIS group and American AIS patients, as well as the current cohort of normal subjects and another cohort with different ethnicity background.

## 3 Results

A total of 657 AIS patients and 248 normal adolescents were enrolled in the present study. In the AIS group, 554 were female and 103 were male. There were 157 females and 91 males. The mean age at presentation for the AIS and normal group were 14.6 ± 2.2 and 15.2 ± 2.1 years (range, 10–18 years), respectively. No significant difference was found in age between the 2 groups (*P* > 0.05).

In the AIS group, 70 (10.6%) patients had variations in the numbers of thoracic and/or lumbar vertebrae: 53 were female and 17 were male. There were 37 (5.6%) patients with abnormal numbers of thoracic vertebrae: 33 patients had 11 thoracic vertebrae and 4 patients had 13. There were 48 (7.3%) patients with abnormal numbers of lumbar vertebrae: 14 patients had 4 lumbar vertebrae and 34 patients had 6. An LSTV was present in 37 patients who had atypical numbers of lumbar vertebrae. Remarkably, 15 (2.3%) patients had atypical numbers of both thoracic and lumbar vertebrae (Table 2). Of the 15 patients, 9 had 13 thoracic vertebrae and 4 lumbar vertebrae; the others had 11 thoracic vertebrae and 6 lumbar vertebrae, including 1 patient with an LSTV.

**Table 2:**
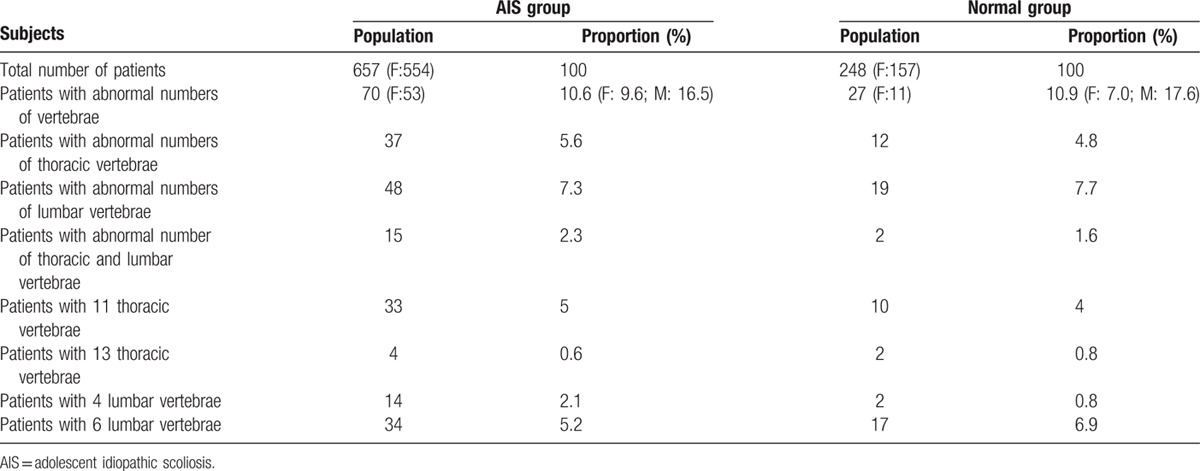
The variations in vertebral number in the AIS and normal group in China.

In the normal group, 27 (10.9%) subjects had variations in the numbers of thoracic and/or lumbar vertebrae: 11 were female and 16 were male. Atypical numbers of thoracic vertebrae were identified in 12 (4.8%) patients: 10 (4%) patients had 11 vertebrae and 2 (0.8%) had 13 vertebrae. Atypical numbers of lumbar vertebrae were identified in 19 (7.7%) patients: 2 (0.8%) patients had 4 lumbar vertebrae and 17 (6.9%) had 6. An LSTV was present in 12 patients who had atypical numbers of lumbar vertebrae. Moreover, 2 (1.6%) of the 248 subjects had atypical numbers of both thoracic and lumbar vertebrae (Table 2). Both of them had 11 thoracic vertebrae, 6 lumbar vertebrae, and an LSTV.

The reports by the radiologist identified 11 patients (1.7%) with a vertebral variation, 9 involving an atypical number of thoracic vertebrae (11 in 7 patients and 13 in the others) and 2 involving an atypical number of lumbar vertebrae (4 in 1 patient and 6 in the other). Due to recount vertebral number through the radiographs preoperatively, no wrong fusion level selection has occurred in the other 59 cases overlooked by the radiologists.

In the Han Chinese population, male subjects had a higher prevalence of variations in the numbers of vertebrae, both in the AIS and normal groups. (*P* = 0.036 and 0.01, respectively) (Table 2). There was no significant difference between the AIS and normal group in the incidence of numerical variations in the numbers of thoracic and/or lumbar vertebrae (all *P* values >0.05). Between Chinese and American patients with AIS, no statistically significant difference was found in terms of the numerical variations in thoracolumbar vertebrae (*P* > 0.05) (Table 3).

**Table 3:**
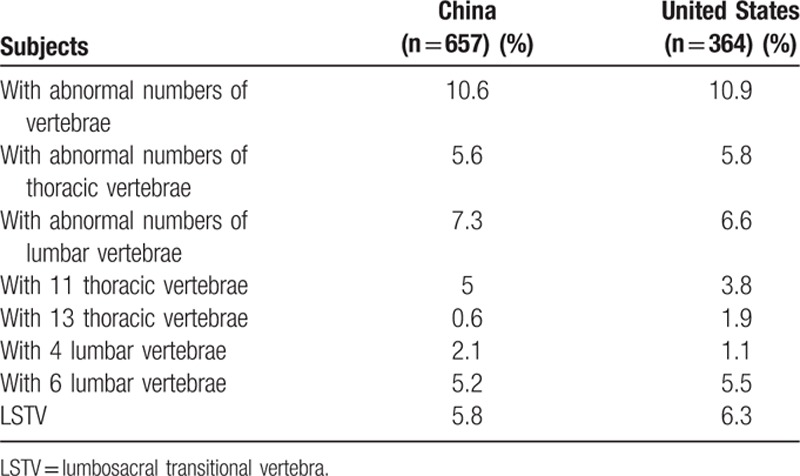
Statistical analysis of variations between Chinese and United States AIS patients.

We compared between female and male subjects in the groups that considered gender, and we found that the incidence of variations in the numbers of vertebrae in male subjects was higher than in female subjects, and there were significant differences among African-American, Inuit, and Chinese populations (*P* = 0.000, 0.001, and 0.001, respectively) (Table 4).

**Table 4:**
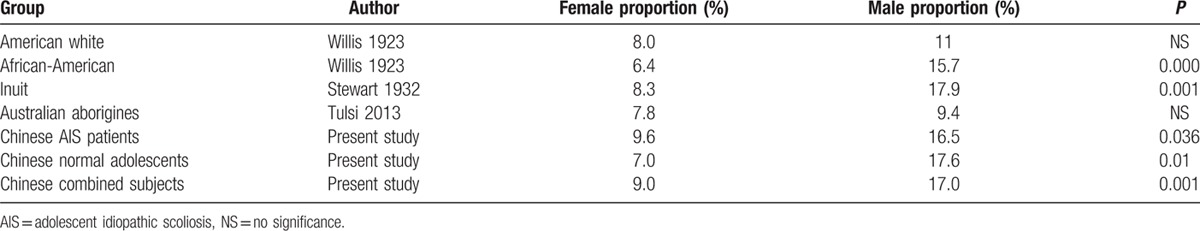
Comparison of proportions of sexes in each group.

## 4 Discussion

In the present study, we found that the variation of number in thoracic–lumbar vertebra in Han Chinese AIS patients and normal adolescents were 10.6% and 10.9%, respectively. The male subjects had a significantly greater variation of vertebral number than female subjects in both the AIS and normal groups (*P* < 0.05), being consistent with previous studies in which the subjects were grouped by gender, and we determined that variations in male subjects were uniformly more frequent than in female subjects (Table 4). This finding appeared to be in contrast to the incidence of AIS, with a female-to-male ratio of 1.2:1.^[2,3]^

Previous studies have noted the importance of vertebral level selection in scoliosis surgery, in which a poor fusion level selection can lead to suboptimal curve correction, coronal and sagittal imbalance, shoulder imbalance, decompensation, and junctional kyphosis.^[19–23]^ As some studies have found, almost 50% of spine surgeons report that they have performed surgery at an incorrect vertebral level during their careers.^[10,11]^ The variable anatomy of patients has been considered a cause in up to 40% of cases.^[10,11,24]^

In white Americans and African-Americans^[14]^ and South African Bantu^[15]^ subjects, authors have counted thoracic–lumbar vertebral numbers, overlooking the presence of 13 thoracic vertebrae and 4 lumbar vertebrae or of 11 thoracic vertebrae and 6 lumbar vertebrae. In Inuit,^[16]^ white American and African-American,^[17]^ and Australian aborigine^[25]^ groups, authors have counted the numbers of presacral vertebrae, calculating only a total of cervical, thoracic, and lumbar vertebrae. These studies could not provide any practical utility for preventing wrong-level surgery due to the data not having been obtained by studying plain films of total spine but rather by studying skeletons in local museums.^[14,16,17,25]^

With the application of standing full-length AP and lateral spine radiographs, Ibrahim et al^[12]^ noted that the frequency of variation in the patients with AIS was approximately 10.4% which was close to our result. However, they did not consider sex variations, nor did they compare the prevalence of variations in the numbers of vertebrae to that in normal subjects.

Some authors suggested that the lack of prospective studies has made it difficult to determine the precise relationship between wrong-level spine surgery and its risk factors.^[26,27]^ An atypical number of vertebrae and the presence of an LSTV could predictably impede an adequate evaluation of spinal anatomy.^[11]^ In the present study, such variations accounted for about 40% of wrong-level discectomies. Mody et al^[10]^ reported that atypical number of lumbar vertebrae and LSTV resulted in most cases of wrong-level surgery, nearly 71% of cases in their series. Other studies that were focused specifically on discectomy surgery in adults have noted the role of an LSTV in wrong-site surgery.^[28,29]^ In the present study, although no wrong-site surgery events have taken place due to deliberate review on the whole-spine plain films, it is a primary point that enumerate vertebrae could reduce the incidence wrong-level spine surgery arising from variations in vertebral number.

To the best of our knowledge, this was the first study to investigate systematically the numerical variations in numbers of thoracic–lumbar vertebrae in subjects both in an AIS group and in a normal group in China, with such a large sample size and with complete radiographs. At the same time, sex, age, and type of vertebral number variation were thoroughly considered.

Several limitations of the present study nevertheless exist. In cases of suspected hypoplastic ribs, obscure vertebrae, and uncertain LSTV on plain films, CT 3-dimensional reconstruction was available to identify the vertebral number. In this study, however, we failed to collect these data from the normal group. Moreover, due to the retrospective design of this study, we could not correlate the variations in the number of vertebrae in the AIS patients with wrong-site surgery or with its effect on correction and balance.

## 5 Conclusion

Among different ethnicity populations, the incidence of variations in thoracic–lumbar number in males was generally higher than that in females. The incidence of variation in patients with AIS in Han Chinese population is up to 10.6%. Instead of depending on the radiologist's report to identify any variation, counting the numbers of thoracic–lumbar vertebrae preoperatively should be performed as a routine procedure.
